# Short-term maternal outcomes after intraoperative administration of prophylactic oxytocin during cesarean sections: a retrospective cohort study with a comparison of different administration protocols

**DOI:** 10.1007/s00404-026-08391-6

**Published:** 2026-03-28

**Authors:** Jakob Thomas Busłowicz, Dörthe Brüggmann, Ammar Al Naimi, Samira Catharina Hoock, Eileen Deuster, Wiebke Schaarschmidt, Anne Kristina Kämpf, Vanessa Neef, Kai Zacharowski, Frank Louwen, Anna Elisabeth Hentrich

**Affiliations:** 1 Department of Obstetrics and Perinatal Medicine, Goethe University Frankfurt, University Hospital, 60590 Frankfurt, Germany; 2 Department of Anaesthesiology, Intensive Care Medicine and Pain Therapy, Goethe University Frankfurt, University Hospital, 60590 Frankfurt, Germany

**Keywords:** Oxytocin, Cesarean section, Elective, Unplanned, Maternal blood loss, Hemoglobin concentration

## Abstract

**Objective:**

The objective of this study was to evaluate the impact of different intraoperative prophylactic oxytocin regimens on maternal blood loss during cesarean section, and to compare effects in procedures performed before versus after onset of labor.

**Methods:**

This retrospective cohort study was conducted at a tertiary care center over 15 years (2006–2021). A total of 1996 cesarean sections were identified, of which 1504 women with complete pre- and postoperative hemoglobin values were included in hemoglobin delta analyses. All 1996 women were considered for descriptive and binary outcome analyses. The study population was stratified into four intraoperative oxytocin exposure groups (0 IU, 3 IU, > 3 up to ≤ 13 IU, and > 13 IU) and further analyzed according to timing before or after onset of labor. The primary outcome was perioperative hemoglobin delta, while secondary outcomes included estimated intraoperative blood loss and binary maternal outcomes such as transfusion, uterine atony, and B-Lynch procedure.

**Results:**

In cesarean sections before onset of labor, very high intraoperative oxytocin doses (> 13 IU) were associated with significantly increased adjusted blood loss (+ 315 ml, p = 0.007), while intermediate doses (+ 270 ml, p = 0.009) also showed higher losses compared with no oxytocin. Hemoglobin decline was greater in the 3 IU and > 3 up to ≤ 13 IU groups, but not in the > 13 IU group. In cesarean sections after onset of labor, women receiving very high doses likewise had significantly higher blood loss (+ 170 ml, p = 0.004), whereas a modest reduction was observed with 3 IU (− 116 ml, p = 0.014). Estimated blood loss did not reliably correlate with hemoglobin decline, and no consistent dose–response benefit of higher oxytocin administration was observed.

**Conclusion:**

Very high intraoperative oxytocin doses were not associated with reduced blood loss and were frequently administered in women at increased bleeding risk, suggesting confounding by indication. These results should therefore be regarded as hypothesis-generating rather than definitive evidence to guide prophylactic dosing.

**Supplementary Information:**

The online version contains supplementary material available at 10.1007/s00404-026-08391-6.

## What does this study add to the clinical work?

**Table:** 

This retrospective cohort suggest that higher intraoperative oxytocin dose during cesarean delivery do not confer a clear dose–response benefit on maternal blood loss and that very high doses are often used in higher risk situations rather than acting protectively. For clinical practice, it supports a cautious, individualized strategy favoring the lowest effective prophylactic oxytocin dose instead of routine high-dose regimens.	

## Introduction

Oxytocin is a peptide hormone secreted by the pituitary gland that performs multiple functions during sexual activity, childbirth, breastfeeding, and maternal bonding [1–4]. In obstetric practice, synthetic oxytocin is widely used for a multitude of medical indications. These include induction of labor [5], augmentation of uterine contractions in the first and second stages of labor, as well as prevention [6] and treatment of postpartum hemorrhage (PPH) [7–9].

Current guidelines vary considerably in their recommendations for prophylactic oxytocin during cesarean section, ranging from unspecified low-dose infusions (German Society of Gynaecology and Obstetrics [10]/American College of Obstetricians and Gynaecologists) to a standardized 5 IU slow intravenous injection (British National Institute of Health and Care Excellence) [11].

Additionally varying recommendations are presented in systematic reviews and meta-analyses regarding oxytocin use for induction, treatment of labor dystocia [12] and treatment or prophylaxis of PPH [8]. These heterogeneous recommendations and references result in a wide range of oxytocin application protocols in clinical practice, but clear and consistent evidence for different peri- and intraoperative oxytocin treatment doses is lacking.

Especially recommendations for the proper use of oxytocin as PPH prophylaxis in women, who deliver by elective CS (before onset of labor) or after the onset of labor, are needed to ensure safe and effective clinical management. In intrapartum CSs, the presence of endogenous oxytocin and the potential prior administration of synthetic oxytocin complicate optimal dosing strategies. Since there is a fine balance between adequate uterine contractions and minimizing adverse maternal and neonatal outcomes [13]—including PPH, cardiovascular effects, nausea and vomiting, impaired lactation and negative birth experience—well-designed studies are necessary to investigate effective and safe prophylactic oxytocin dosing in patients undergoing CS.

In this study, we aimed to determine the necessity and optimal protocol for prophylactic synthetic oxytocin administration during CS in preventing short-term maternal complications. Specifically, we assessed the association between intraoperative oxytocin exposure and maternal blood loss in nulliparous women delivering a live singleton at term. We hypothesized that higher prophylactic intraoperative oxytocin doses would not be associated with lower maternal blood loss.

## Materials and methods

### Study design

We conducted a retrospective cohort study investigating the association between intraoperative prophylactic oxytocin administration and maternal blood loss during CS. All CS performed between 2006 and 2021 at a tertiary perinatal center were screened for inclusion. Eligible participants were nulliparous women aged 18 years and older who delivered a live singleton at term (from 37 weeks onwards at the day of delivery) without major malformation. Maternal or fetal indications for the CS included maternal request, prolonged or arrest of labor, or non-reassuring fetal heart rate tracing. Exclusion criteria comprised relevant fetal malformations, multiples and maternal comorbidities such as coagulopathies, hemoglobinopathies or polyhydramnios.

The final study population was stratified into four oxytocin exposure groups: 0 IU, 3 IU, > 3 IU up to ≤ 13 IU, and > 13 IU. Maternal blood loss was evaluated using perioperative hemoglobin delta as well as the estimated intraoperative blood loss. Furthermore, a secondary analysis stratified whether a CS was before onset of labor (elective or unplanned) or performed after onset of labor.

### Group analysis

Based on clinical relevance, we further stratified the cohort by timing of the CS: (1) the CS was before onset of labor and before any progress was made in the birth process or (2) CS after onset of labor (e.g., first or second stage of labor, defined by diagnosis of labor arrest or fetal/maternal compromise during the first and second stage of labor).

### Oxytocin exposure

In our institution, oxytocin is not routinely administered for labor induction or during the first stage of labor. Patients therefore received oxytocin only during advanced labor and/or during CS. Intrapartum oxytocin administration for augmentation was systematically documented in the standardized partogram and modeled as a separate covariate in multivariable regression analyses.

Intraoperative oxytocin exposure was defined as the cumulative dose administered from delivery of the neonate until the end of surgery, calculated as the sum of all boluses and infusions documented in the operative and anesthesia records. For example, a total of 13 IU corresponded to a 3 IU slow intravenous bolus followed by a 10 IU infusion over several minutes.

During the study period, the institutional intravenous oxytocin protocol was gradually reduced in multiple steps from 26 IU to none. Deviations from the valid protocol at the time were permitted only if the obstetric surgeon considered uterine contraction insufficient. Calendar year was therefore included as a covariate in regression models to account for these institutional changes.

Patients were stratified into four intraoperative dose groups:

1. IU (no exposure group).
2. 3 IU
3. > 3 up to ≤ 13 IU.
4. > 13 IU.

Analyses were further stratified by timing of CS before versus after onset of labor to account for potential receptor desensitization in laboring women.

Women with postpartum hemorrhage, transfusion, uterine atony, administration of sulprostone, or B-Lynch procedures were not excluded; these events were systematically documented and are presented as outcomes in the results tables.

Due to the retrospective design, intraoperative clinical decisions (e.g., administration of higher oxytocin doses in cases of uterine atony or bleeding) could not be fully controlled for, which may introduce confounding by indication.

### Data collection

We extracted all relevant variables from the electronic health records, patients’ charts, and federal perinatal database *Perinatalerhebung Hessen*, which includes standardized obstetric and neonatal outcome data in the state of Hessen. We analyzed the data between July 1, 2023, and September 30, 2024.

We obtained approval of the local ethics committee to conduct the study (protocol #2022–930). Outcome data were routinely collected during standard medical care and analyzed in an anonymous and retrospective form. Hence, no specific written consent signed by the patients was requested.

The following maternal and neonatal variables were assessed: maternal age, birthweight, relevant comorbidities including preeclampsia/eclampsia, diabetes as well as intrauterine inflammation/infection (triple I).

### Outcomes

The primary outcome was perioperative hemoglobin decline, defined as the difference between the last preoperative hemoglobin measurement and the first postoperative hemoglobin measurement obtained within 24 h after cesarean delivery. Preoperative hemoglobin testing was routinely performed in women with identified obstetric or medical risk factors, planned cesarean delivery, or anticipated perioperative risk. In contrast, women admitted for planned vaginal birth without clinical risk factors did not routinely undergo immediate preoperative hemoglobin testing according to institutional standards. Consequently, missing hemoglobin values primarily reflect structured clinical workflow rather than random data loss. Hemoglobin-based analyses therefore represent the subgroup in whom laboratory testing was clinically indicated. Consequently, perioperative hemoglobin decline could only be calculated in patients with both measurements available. Complete perioperative hemoglobin values were available in 1504 women, and hemoglobin-based analyses were restricted to these complete cases. Missing hemoglobin values therefore reflect routine clinical practice rather than study-specific data loss.

Estimated intraoperative blood loss, determined by visual estimation of the surgical team and documented in the operative report, was analyzed as a secondary exploratory outcome. Estimated intraoperative blood loss was available in 1970 women.

### Statistical analysis

For continuous normally distributed variables, group comparisons were made using analysis of variance (ANOVA); for categorical variables, Chi-square tests were applied. Linear regression models (unadjusted and multivariable adjusted for maternal age, BMI, infant birthweight, type of anesthesia, intrapartum oxytocin, calendar year, macrosomia—defined as birthweight ≥ 4000 g, and perioperative fluid administration) were used to examine the association of oxytocin dose categories with hemoglobin delta and blood loss. Total intraoperative crystalloid volume (ml) was included as a continuous covariate in multivariable regression models. For binary outcomes (e.g., uterine atony, transfusion, blood loss ≥ 1000 ml, B-Lynch suture, and hysterectomy), logistic regression models were applied including the same covariates. In several analyses, adjusted models yielded different effect estimates than unadjusted comparisons, underlining the importance of accounting for potential confounding.

Multivariable linear regression models were used to evaluate associations between oxytocin dose groups and outcomes. Adjusted models included maternal age, neonatal birthweight, anesthesia type, timing of labor onset, macrosomia, maternal comorbidities, and total intraoperative crystalloid volume.

Due to the retrospective nature of the study and routine clinical data collection, missing values were present for some covariates and hemoglobin measurements. Adjusted analyses were therefore performed using complete-case data. No imputation was performed, as missing hemoglobin values primarily reflected the absence of routine laboratory testing rather than study-specific data loss. Due to the retrospective design, intraoperative clinical decisions (e.g., administering more oxytocin in cases of uterine atony or bleeding) could not be fully controlled for, which may introduce confounding by indication. To evaluate potential bias due to missing laboratory values, we compared women with and without complete perioperative hemoglobin measurements; results are provided in Supplementary Table S1.

Estimated intraoperative blood loss was analyzed as a secondary exploratory outcome due to the known limitations of visual estimation.

The cut-off value for statistical significance was set at *p* < 0.05, and all statistical analyses were conducted using Stata version 18. The study was conducted and reported in accordance with the STROBE guidelines for observational cohort studies.

## Results

**Fig. 1:**
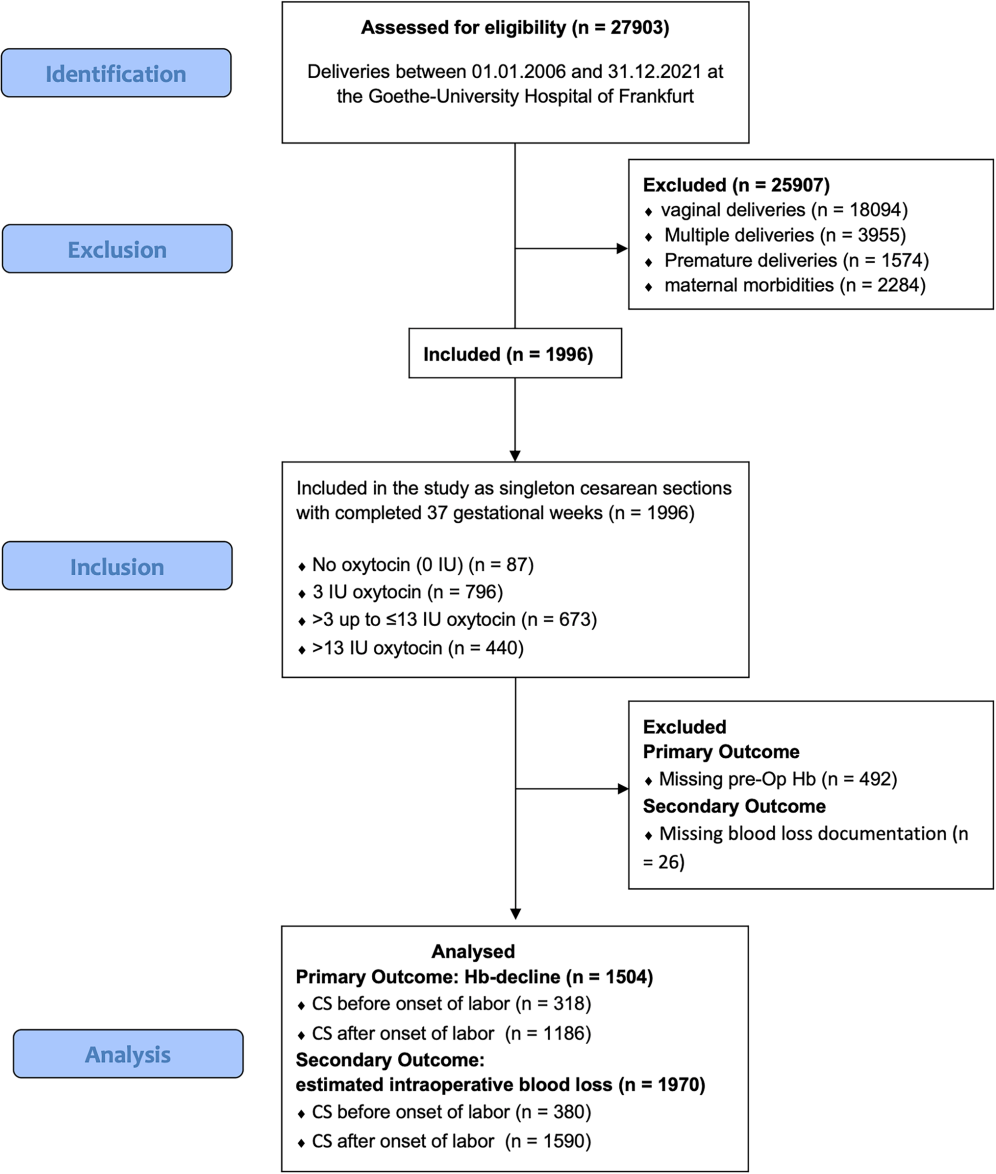
Flow diagram: A total of 1996 CS were identified during the study period. Of these, 26 cases lacked documentation of intraoperative blood loss and were excluded from blood loss analyses, leaving 1970 women for analyses of estimated blood loss. Separately, 492 women from the overall cohort had no preoperative hemoglobin value available. The final analytic cohort for hemoglobin-based outcomes therefore comprised 1504 complete cases

A total of 1996 cesarean deliveries were included in the overall cohort. Estimated intraoperative blood loss was available in 1970 women. Complete perioperative hemoglobin measurements were available in 1504 women, and analyses involving hemoglobin decline were restricted to this complete-case cohort. Preoperative hemoglobin was missing in 492 women (24.7%), who were therefore excluded from Hb-delta analyses but retained for descriptive and binary outcome analyses (complete-case approach, no imputation). Baseline characteristics of women with and without perioperative hemoglobin measurements are shown in Supplementary Table S1. For analysis of intraoperative blood loss, 1970 women were included; 26 surgical records lacked blood loss documentation. Among the 1504 women with complete perioperative hemoglobin values, 318 underwent cesarean delivery before onset of labor and 1186 after onset of labor. Oxytocin exposure groups were as follows: no oxytocin (*n* = 87), 3 IU (*n* = 796), > 3 up to ≤ 13 IU (*n* = 673), and > 13 IU (*n* = 440). See Fig. 1 for study flow.

### Demographics

The baseline demographic characteristics are presented in Table 1. No statistically significant differences were found between the oxytocin exposure groups regarding maternal age (*p* = 0.34), BMI prior to gestation (*p* = 0.84), gestational age (*p* = 0.13), relevant comorbidities, or whether birth induction was performed (*p* = 0.13). Newborns had an average birthweight of 3384 g, with statistically significant differences between the oxytocin exposure groups. Infants in the 3 IU (3351 g), > 3 up to ≤ 13 IU (3409 g), and > 13 IU (3420 g) groups had significantly higher birthweights compared with the no-oxytocin group (p = 0.02).

**Table 1:** Demographic characteristics

Variable	No oxytocin (*n* = 87)	3 IU oxytocin (*n* = 796)	> 3 up to ≤ 13 IU oxytocin (*n* = 673)	> 13 IU oxytocin (*n* = 440)	*p*
Age, years (mean ± SD)	32.6 (5.0)	32.3 (4.7)	32.2 (5.0)	31.8 (5.1)	0.34
BMI prior, kg/m^2^ (mean ± SD)	24.4 (5.2)	24.5 (5.4)	24.6 (7.3)	24.0 (4.5)	0.84
Gestational age, weeks (mean ± SD)	39.3 (1.3)	39.6 (1.3)	39.6 (1.2)	39.5 (1.3)	0.13
Induction of labor, n (%)	24 (27.6%)	237 (29.8%)	225 (33.4%)	119 (27.0%)	0.12
Elective CS, n (%)	16 (18.4%)	197 (24.8%)	80 (11.9%)	90 (20.5%)	< 0.001
Augmentation, n (%)	4 (13.8%)	3 (1.0%)	6 (4.0%)	32 (32.3%)	< 0.001
Type of anesthesia, n (%)					< 0.001
Epidural	35 (40.2%)	316 (39.8%)	363 (54.4%)	227 (51.9%)	
Spinal	44 (50.6%)	426 (53.7%)	226 (33.9%)	168 (38.4%)	
General	8 (9.2%)	43 (5.4%)	72 (10.8%)	39 (8.9%)	
Preoperative Hb, g/dl (mean ± SD)	12.23 (1.08)	12.17 (1.3)	12.02 (1.09)	12.11 (1.04)	0.143
Birthweight, g (mean ± SD)	3306.6 (527.1)	3351.3 (493.9)	3409.7 (493.8)	3420.4 (488.0)	0.020
Labor arrest (2nd stage), n (%)	10 (11.5%)	112 (14.1%)	140 (20.8%)	86 (19.5%)	0.002
Intrauterine infection (triple I), n (%)	4 (4.6%)	8 (1.0%)	35 (5.2%)	33 (7.5%)	< 0.001
Diabetes, n (%)	9 (10.3%)	123 (15.5%)	112 (16.6%)	66 (15.0%)	0.47
Preeclampsia, n (%)	3 (3.4%)	26 (3.3%)	21 (3.1%)	16 (3.6%)	0.97

The mean preoperative hemoglobin level was 12.21 g/dL (SD 1.12) in women undergoing CS before onset of labor, and 12.09 g/dL (SD 1.08) in those after onset of labor. This difference was not statistically significant (*p* = 0.063). The volume of infused crystalloid fluids differed significantly between groups: women delivered by CS before onset of labor received on average 1025 ml, whereas those after onset of labor received 897 ml (*p* < 0.001).

### Primary outcome: intraoperative Hb difference

Among patients who underwent CS before the onset of labor and did not receive oxytocin, the perioperative decrease in hemoglobin levels was statistically significant, reflecting the expected physiological decline. The three oxytocin exposure groups (3 IU, > 3 up to ≤ 13 IU, and > 13 IU) were then compared with the no-oxytocin reference group to assess additional effects on hemoglobin decline. In unadjusted comparisons across oxytocin dose groups, no consistent differences in hemoglobin delta were observed. In the fully adjusted model (including maternal age, BMI, birthweight, type of anesthesia, intrapartum oxytocin exposure, calendar year, macrosomia, and perioperative fluid administration), women receiving 3 IU (β = − 1.463, *p* = 0.035) and > 3 up to ≤ 13 IU (β = − 1.653, *p* = 0.050) showed significantly greater hemoglobin declines compared with the no-oxytocin group. Very high doses (> 13 IU) were also associated with a larger decline (β = − 1.105), although this result did not reach statistical significance (*p* = 0.233; Table 2).

**Table 2:** Maternal outcomes—before onset of labor

Outcome	Dose group	Mean diff. ± SE	95% CI	*p*
Hb delta (g/dL)	3 IU	−0.61 ± 0.29	−1.18 to −0.04	0.034
Crude	> 3 up to ≤ 13 IU	−0.27 ± 0.30	−0.86 to +0.32	0.372
	> 13 IU	−0.55 ± 0.30	−1.14 to +0.04	0.065
	3 IU	−1.46 ± 0.68	−2.79 to −0.13	0.035
Adjusted	> 3 up to ≤ 13 IU	−1.65 ± 0.83	−3.28 to −0.02	0.050
	> 13 IU	−1.11 ± 0.92	−2.91 to 0.69	0.233
Estimated blood loss (ml)	3 IU	−58 ± 40	−136.4 to +20.4	0.145
Crude	> 3 up to ≤ 13 IU	−89 ± 42	−171.3 to −6.7	0.033
	> 13 IU	−149 ± 42	−231.3 to −66.7	< 0.001
	3 IU	+ 79 ± 89	−95.4 to +253.4	0.379
Adjusted	> 3 up to ≤ 13 IU	+ 270 ± 101	+72.0 to +468.0	0.009
	> 13 IU	+ 315 ± 114	+91.6 to +538.4	0.007

Hb delta and estimated blood loss: linear regression (unadjusted and multivariable). Adjusted models controlled for maternal age, BMI, infant birthweight, type of anesthesia, intrapartum oxytocin exposure, and calendar year. Reference group: 0 IU oxytocin

In women undergoing CS after the onset of labor without intraoperative oxytocin, a significant perioperative hemoglobin decline was also documented, consistent with the group before onset of labor. In unadjusted comparisons across oxytocin dose groups, no significant differences in hemoglobin delta were observed. In the fully adjusted model (including maternal age, BMI, birthweight, type of anesthesia, intrapartum oxytocin exposure, calendar year, macrosomia, and perioperative fluid administration), oxytocin dose was not significantly associated with hemoglobin delta. Effect estimates were small and did not reach statistical significance (3 IU: β = + 0.269, *p* = 0.318; > 3 up to ≤ 13 IU: β = + 0.487, *p* = 0.067; > 13 IU: β = + 0.394, *p* = 0.191; Table 3).

**Table 3:** Maternal outcomes—after onset of labor

Outcome	Dose group	Mean diff. ± SE	95% CI	*p*
Hb delta (g/dL)	3 IU	−0.01 ± 0.15	−0.30 to +0.28	0.957
Crude	> 3 up to ≤ 13 IU	+ 0.19 ± 0.15	−0.10 to +0.48	0.205
	> 13 IU	+ 0.20 ± 0.15	−0.09 to +0.49	0.197
	3 IU	+ 0.27 ± 0.27	−0.26 to +0.80	0.318
Adjusted	> 3 up to ≤ 13 IU	+ 0.49 ± 0.26	−0.02 to +1.00	0.067
	> 13 IU	+ 0.39 ± 0.30	−0.20 to +0.98	0.191
Estimated blood loss (ml)	3 IU	−20 ± 31	−80.8 to +40.8	0.529
Crude	> 3 up to ≤ 13 IU	+ 40 ± 31	−20.8 to +100.8	0.203
	> 13 IU	−20 ± 32	−82.7 to +42.7	0.540
	3 IU	−116 ± 47	−208.1 to −23.9	0.014
Adjusted	> 3 up to ≤ 13 IU	−4.8 ± 47	−96.9 to +87.3	0.919
	> 13 IU	+ 170 ± 58	+56.3 to +283.7	0.004

Hb delta and estimated blood loss: linear regression (unadjusted and multivariable). Adjusted models controlled for maternal age, BMI, infant birthweight, type of anesthesia, intrapartum oxytocin exposure, and calendar year. Reference group: 0 IU oxytocin

### Secondary outcomes: documented blood loss (in ml)

In women undergoing CS before onset of labor, unadjusted analyses suggested lower mean blood loss with increasing oxytocin doses (3 IU: − 58 ml, *p* = 0.145; > 3 up to ≤ 13 IU: − 89 ml, *p* = 0.033; > 13 IU: − 149 ml, *p* < 0.001) compared with the no-oxytocin group. However, in the fully adjusted model, this pattern reversed: very high intraoperative doses were associated with significantly higher blood loss (> 13 IU: + 315 ml, *p* = 0.007), while intermediate doses also showed a significant increase (+ 270 ml, *p* = 0.009). The 3 IU dose did not differ significantly from the reference group (+ 79 ml, *p* = 0.379; Table 2). Adjusted analyses included fewer patients due to missing covariate data, as all covariates were required for inclusion in multivariable models.

The findings from Tables 2 and 3 represent the primary analyses, whereas the sensitivity analyses in Table 4 mainly confirmed the overall pattern, albeit with attenuated associations.

**Table 4:** Sensitivity analysis

Dose group	Before onset of labor [mean diff. ± SE (CI 95%), adj.]	*p*	After onset of labor [mean diff. ± SE (CI 95%), adj.]	*p*
3 IU	+ 44 ± 99 ml (−150.0 to +238.0)	0.655	−79 ± 28 ml (−133.9 to −24.1)	0.005
> 3 up to ≤ 13 IU	+ 130 ± 108 ml (−81.7 to +341.7)	0.230	−15 ± 28 ml (−69.9 to +39.9)	0.590
> 13 IU	-37 ± 103 ml (−238.9 to +164.9)	0.723	+ 34 ± 36 ml (−36.6 to +104.6)	0.333

Sensitivity analysis: exclusion of cases with transfusion, B-Lynch procedure, hysterectomy, or severe atony. Results from multivariable linear regression adjusted for maternal age, BMI, infant birthweight, type of anesthesia, intrapartum oxytocin exposure, and calendar year. Reference group: 0 IU oxytocin

Among women undergoing CS after onset of labor, unadjusted analyses showed no significant differences across oxytocin groups. In the fully adjusted model, women receiving very high doses experienced significantly higher blood loss (> 13 IU: + 170 ml, *p* = 0.004), while those given 3 IU had a modest but statistically significant reduction (− 116 ml, *p* = 0.014). Intermediate doses were not significantly associated with blood loss (− 5 ml, *p* = 0.919; Table 3). Sensitivity analyses excluding women with transfusion, B-Lynch, or hysterectomy showed attenuated associations, with a reduction in the 3 IU group (− 79 ml, *p* = 0.005) and no significant effect for > 13 IU (+ 34 ml, *p* = 0.333; Table 4).

In analyses of binary outcomes, uterine atony (*p* = 0.034), B-Lynch procedures (*p* = 0.044), and hysterectomy (*p* = 0.044) were significantly associated with oxytocin dose categories, whereas transfusion showed a statistical trend (*p* = 0.068). After multivariable adjustment, associations for postpartum hemorrhage ≥ 1000 ml were not statistically significant (Table 5).

**Table 5:** Atony, transfusion, B-Lynch, hysterectomy: logistic regression

Complication	No Oxytocin	3 IU	> 3 up to ≤ 13	> 13 IU	*p* (crude)	*p* (adjusted)
Atony, n (%)	0 (0.0)	2 (0.3)	41 (6.1)	13 (3.0)	< 0.001	0.034
Transfusion, n (%)	0 (0.0)	4 (0.5)	12 (1.8)	2 (0.5)	0.029	0.068
B-Lynch, n (%)	0 (0.0)	0 (0.0)	17 (2.5)	7 (1.6)	< 0.001	0.044
Hysterectomy, n (%)	0 (0.0)	1 (0.1)	1 (0.1)	0 (0.0)	0.860	0.044

## Discussion

Given these findings, oxytocin dosing should be considered not only in relation to uterine contractility and blood loss but also within the broader context of maternal safety.

Oxytocin is known to exert dose-dependent cardiovascular effects such as hypotension and tachycardia. In addition, repeated or high cumulative exposure may contribute to desensitization of myometrial oxytocin receptors [14–18]. These physiological aspects emphasize the need to evaluate prophylactic oxytocin administration with respect to both efficacy and safety [19, 20], advocating for a restrained dosing strategy during cesarean delivery.

Carvalho et al. [20] investigated the required bolus dose in a prospective blinded trial of elective CS and demonstrated that the amount necessary for adequate uterine contraction could be significantly reduced. Similarly, the trials performed by Seagraves et al. [21] and Nagai et al. [22] showed that prophylactic oxytocin doses can be substantially lowered, although the absolute doses used in those studies were markedly higher than in our cohort. These findings are consistent with the recommendations by Stephens and Bruessel [23], who concluded that low-dose regimens (0.3–1 IU bolus followed by 5–10 IU/h infusion) are effective for prophylaxis in elective CS and sufficient to prevent atony.

The required effective oxytocin dose (ED_90_) was investigated by George et al. [24] and found to be 15 IU administered over 1 h, which is higher than the dose ranges observed in our cohort (> 3 up to ≤ 13 IU). A distinctive feature of our study is the inclusion of a patient group who received no oxytocin at all. Moreover, while most previous studies reported maternal blood loss in milliliters and the occurrence of postpartum hemorrhage endpoints, our study additionally evaluated perioperative hemoglobin decline, thereby providing a complementary and more objective marker of blood loss.

Intraoperative blood loss estimates based on visual assessment and operative reports did not demonstrate a significant difference across oxytocin regimes. Other studies resulted in similar findings [25, 26]. Stålberg et al. [27] found a slightly higher total blood loss in the reduced oxytocin group when including the continued bleeding up to 2 h postoperatively. It must be noted that most studies compare a vast amount of different administration protocols, exclusion criteria differ significantly, and endpoints are inconsistent. In our fully adjusted analyses, very high intraoperative doses (> 13 IU) were associated with significantly higher blood loss in CS before onset of labor (+ 315 ml, p = 0.007), while intermediate doses also showed a significant increase (+ 270 ml, p = 0.009). In CS after onset of labor, women receiving very high doses again had significantly higher blood loss (+ 170 ml, p = 0.004), whereas those given 3 IU experienced a modest but significant reduction (− 116 ml, p = 0.014). Intermediate doses were not significantly different from the reference group. These findings were attenuated in sensitivity analyses that excluded cases with transfusion, B-Lynch procedures, or hysterectomy, where the association for > 13 IU was no longer significant and only the 3 IU group retained a modest reduction. This highlights the importance of accounting for confounding variables, including maternal age, BMI, birthweight, anesthesia, intrapartum oxytocin exposure, calendar year, macrosomia, and perioperative fluid administration.

A key finding of our study is the discordance between objectively measured perioperative hemoglobin decline and subjectively documented intraoperative blood loss. Women undergoing CS before onset of labor without oxytocin exposure exhibited a greater Hb drop that was not reflected in operative reports, underscoring the limited reliability of intraoperative blood loss documentation. This illustrates the inaccuracy of visual estimation methods in clinical practice. Visual assessment of blood loss is generally considered flawed and prone to underestimation [28], and several studies have shown that obstetricians tend to underestimate blood loss [29], while similar limitations have been demonstrated in other surgical fields [28, 30]. These findings emphasize the value of including objective measures such as Hb-delta and highlight the need to establish more reliable methods of blood loss quantification, particularly in high-risk scenarios.

The prophylactic administration of oxytocin did not reduce maternal blood loss or hemoglobin decline in CS after onset of labor. Previous publications had recommended higher prophylactic doses for intrapartum CS [23].

However, in our adjusted main analyses, women receiving 3 IU showed a modest but statistically significant reduction in blood loss (− 116 ml, *p* = 0.014), whereas very high doses (> 13 IU) were associated with a significantly increased blood loss (+ 170 ml, *p* = 0.004). Intermediate doses did not differ from the no-oxytocin group. Sensitivity analyses excluding women with transfusion, B-Lynch procedures, or hysterectomy showed attenuated associations, with only the 3 IU group retaining a modest reduction (− 79 ml, *p* = 0.005) and no significant effect for > 13 IU (+ 34 ml, *p* = 0.333).

With respect to hemoglobin decline, oxytocin dose was not independently associated with perioperative changes in either subgroup. Although receptor desensitization due to concurrent endogenous and exogenous oxytocin exposure has been hypothesized [19, 31, 32], no clinically relevant effects on hemoglobin delta were evident in our data.

In the literature, most oxytocin protocols have been investigated only in elective CS, owing to concerns about increased blood loss in intrapartum procedures [19]. The study by Lavoie et al. [31] determined the ED_90_ requirement of up to 40 IU of oxytocin per hour for prophylactic infusion during CS after prior labor induction with oxytocin. Similarly, a ninefold increase in the oxytocin bolus dose was required for adequate uterine contraction after cesarean delivery following labor arrest in previously oxytocin-exposed women [19, 32] In our clinic, however, oxytocin augmentation during labor or induction is used cautiously and infrequently. This practice may at least partly contribute to an increased sensitivity to exogenous oxytocin in our cohort.

A clear strength of this study is the inclusion of a relatively large number of patients who underwent CS without any oxytocin administration (*n* = 87). For all patients, detailed documentation of prophylactic oxytocin units was available. To gain further insights, we conducted a sensitivity analysis excluding women with massive blood loss or documented atony, thereby minimizing the influence of therapeutic oxytocin use and allowing a more precise investigation of the prophylactic effect. In addition, by stratifying our study groups according to the timing of CS in relation to labor, we were able to include a large cohort after the onset of labor, a group underrepresented in most previous studies. We also explicitly accounted for potential confounding by indication, recognizing that higher intraoperative doses may have been administered in response to bleeding or uterine atony rather than as true prophylaxis.

The data suggest a dose–response relationship between intraoperative oxytocin administration and perioperative blood loss. A key methodological limitation of this study is confounding by indication. In routine clinical practice, higher intraoperative oxytocin doses were commonly administered to women with insufficient uterine tone or an increased risk of bleeding. Thus, the observed association between higher oxytocin doses and greater blood loss likely reflects underlying clinical risk rather than a direct causal effect of oxytocin. Accordingly, our findings should be interpreted as hypothesis‑generating and require confirmation in prospective randomized trials. Established evidence supports that low-dose oxytocin regimens with infusion are adequate for prophylaxis in cesarean delivery [23].

Several limitations should be considered. First, perioperative hemoglobin measurements were not available for all women, as preoperative testing was performed less consistently in urgent intrapartum procedures. Consequently, analyses of hemoglobin decline were restricted to complete cases, which may introduce selection bias. A comparison of women with and without perioperative hemoglobin values showed similar maternal age, BMI, and visually estimated intraoperative blood loss, arguing against systematic exclusion of women with excessive bleeding. Differences were mainly observed in birthweight, cesarean section type, and oxytocin dose distribution, indicating that missing hemoglobin values were largely related to clinical workflow and procedural urgency. Nevertheless, because missingness was associated with these clinical factors, residual selection bias cannot be excluded. Second, adjusted regression models were based on complete-case data and therefore included fewer patients than crude analyses. This reduction in sample size reflects availability of complete covariate data rather than study-specific exclusions. Differences between crude and adjusted estimates likely reflect both adjustment for clinical confounders and differences in patient characteristics within the complete-case subset. These findings highlight the importance of interpreting adjusted analyses in the context of complete-case methodology. These findings should therefore be interpreted cautiously.

Third, estimated intraoperative blood loss was determined by visual assessment and is subject to measurement variability. Therefore, perioperative hemoglobin decline was defined as the primary outcome, as it represents a more objective surrogate measure of blood loss.

A limitation of our dataset is that perioperative side effects of oxytocin, such as hypotension, tachycardia, nausea, or myocardial ischemia, were not systematically documented. These safety parameters represent clinically relevant consequences of oxytocin administration and should be addressed in future prospective studies.

While significant differences in severe maternal events such as uterine atony, transfusion, and B-Lynch procedures were observed between oxytocin dose groups, these outcomes occurred only in a small proportion of patients. Thus, although relevant for clinical safety considerations, they do not alter the overall interpretation that intraoperative oxytocin dosing was not associated with reductions in average blood loss or hemoglobin decline. The observed associations with atony, B-Lynch, and hysterectomy likely reflect confounding by indication, as higher doses were administered in response to established bleeding rather than as true prophylaxis. The findings of this study indicate that low-dose oxytocin regimens (3 IU) appear adequate in selected clinical settings, while very high doses were associated with increased maternal blood loss. These results are hypothesis-generating and require confirmation in prospective trials. They support careful evaluation of current dosing practices and reinforce the principle of using the lowest effective prophylactic dose during cesarean delivery.

## Conclusion

In this large retrospective study, intraoperative oxytocin dosing was associated with perioperative blood loss and hemoglobin decline, with effects differing between CS before and after onset of labor. In procedures before onset of labor, very high doses (> 13 IU) were linked to significantly increased adjusted blood loss (+ 315 ml), while in CS after onset of labor, very high doses were associated with increased adjusted blood loss, most likely reflecting confounding by indication (+ 170 ml), whereas a modest reduction was observed with 3 IU (− 116 ml). Importantly, the associations with uterine atony, B-Lynch procedures, and hysterectomy likely reflect confounding by indication, as higher doses were often administered in response to bleeding.

While prospective studies are needed to validate these observations, the results support the use of the lowest effective prophylactic oxytocin dose during cesarean delivery. Although preliminary, these findings may contribute to future efforts aimed at refining guideline recommendations on prophylactic oxytocin use.

## Supplementary Information

Below is the link to the electronic supplementary material.

Supplementary file1 (DOCX 17 KB)

## Data Availability

No datasets were generated or analyzed during the current study.
