# Supplementary material for: Short-term maternal outcomes after intraoperative administration of prophylactic oxytocin during cesarean sections: a retrospective cohort study with a comparison of different administration protocols
Source: Arch Gynecol Obstet. 2026 Mar 28;313(1):146. doi: 10.1007/s00404-026-08391-6 (PMC13032929; doi:10.1007/s00404-026-08391-6)
Supplement: Supplementary file 1 — Supplementary file1 (DOCX 17 KB) [file 404_2026_8391_MOESM1_ESM.docx]

| **Variable** | **Complete Hb available (n = 1504)** | **Missing Hb (n = 492)** | **p‑value** |
| --- | --- | --- | --- |
| Age, years, mean ± SD | 32.2 ± 5.0 | 32.2 ± 4.8 | 0.92 |
| BMI, kg/m², mean ± SD | 24.8 ± 6.20 | 24.2 ± 4.8 | 0.29 |
| Birthweight, g, mean ± SD | 3372.6 ± 495.6 | 3417.4 ± 505.0 | 0.082 |
| Gestational week | 39.5 (1.3) | 39.8 (1.2) | <0.001 |
| Estimated intraoperative blood loss, ml, mean ± SD | 438.71 ± 227.91 | 446.40 ± 234.52 | 0.55 |
| Oxytocin dose categories,  n (%) |  |  | <0.001 |
| – 0 IU | 69 (4.6) | 18 (3.7) |  |
| – 3 IU | 539 (37.1) | 257 (55.6) |  |
| – >3 to ≤13 IU | 484 (32.2) | 189 (41.4) |  |
| More than 13 IU | 412 (27.4) | 28 (5.7) |  |
| Type of anesthesia, n (%) |  |  | 0.93 |
| – 1 = Epidural anesthesia (PDA) | 716 (47.2) | 239 (48.6) |  |
| – 2 = Spinal anesthesia (SPA) | 660 (43.5) | 216 (43.8) |  |
| – 3 = General anesthesia (ITN) | 128 (8.4) | 37 (7.5) |  |
| Sectio type, n (%) |  |  | <0.001 |
| – 1 = Primary (elective) CS | 330 (21.6) | 66 (13.4) |  |
| – 2 = Secondary (intrapartum) CS | 1174 (78.4) | 426 (86.6) |  |
| diabetes | 234 (15.3) | 77 (15.6) | 0.90 |

**Supplementary Table 1: Comparison of baseline characteristics between women with complete and missing perioperative hemoglobin measurements** Continuous variables were compared using t-tests or Wilcoxon tests, and categorical variables using chi-square tests.
